# Exploring somatic mutations in *BRAF*, *KRAS*, and *NRAS* as therapeutic targets in Saudi colorectal cancer patients through massive parallel sequencing and variant classification

**DOI:** 10.3389/fphar.2024.1498295

**Published:** 2024-11-20

**Authors:** Thamer Abdulhamid Aljuhani, Noor Ahmad Shaik, Rahaf Talal Alqawas, Rana Y. Bokhary, Mahmoud Almutadares, Hadiah Bassam Al Mahdi, Nuha Al-Rayes, Ashraf AbdulRahman El-Harouni, Ramu Elango, Babajan Banaganapalli, Zuhier Ahmad Awan

**Affiliations:** ^1^ Department of Genetic Medicine, Faculty of Medicine, King Abdulaziz University, Jeddah, Saudi Arabia; ^2^ Princess Al-Jawhara Al-Brahim Centre of Excellence in Research of Hereditary Disorders, King Abdulaziz University, Jeddah, Saudi Arabia; ^3^ Molecular Diagnostic Laboratory at King Abdulaziz University Hospital, Jeddah, Saudi Arabia; ^4^ Department of Pathology, Faculty of Medicine, King Abdulaziz University, Jeddah, Saudi Arabia; ^5^ Research and Development Unit, Alborg Diagnostics, Jeddah, Saudi Arabia; ^6^ Department of Medical Laboratory Technology, Faculty of Applied Medical Sciences, King Abdulaziz University, Jeddah, Saudi Arabia; ^7^ Department of Clinical Biochemistry, Faculty of Medicine, King Abdulaziz University, Jeddah, Saudi Arabia

**Keywords:** somatic mutations, BRAF V600E, TruSight tumor 15 panel, targeted drug therapy, colorectal cancer

## Abstract

**Background:**

Colorectal cancer (CRC) is the leading cancer among Saudis, and mutations in *BRAF*, *KRAS*, and *NRAS* genes are therapeutically significant due to their association with pathways critical for cell cycle regulation. This study evaluates the prevalence and frequency of somatic mutations in these actionable genes in Saudi CRC patients and assesses their pathogenicity with bioinformatics methods.

**Methodology:**

The study employed the TruSight Tumor 15 next-generation sequencing (NGS) panel on 86 colorectal cancer (CRC) samples to detect somatic mutations in *BRAF*, *KRAS*, and *NRAS* genes. Bioinformatic analyses of NGS sequences included variant annotation with ANNOVAR, pathogenicity prediction, variant reclassification with CancerVar, and extensive structural analysis. Additionally, molecular docking assessed the binding of Encorafenib to wild-type and mutant *BRAF* proteins, providing insights into the therapeutic relevance of pathogenic variants.

**Results:**

Out of 86 tumor samples, 40 (46.5%) harbored somatic mutations within actionable genes (*BRAF*: 2.3%, *KRAS*: 43%, *NRAS*: 2.3%). Fourteen missense variants were identified (*BRAF*: n = 1, *KRAS*: n = 11, *NRAS*: n = 2). Variants with strong clinical significance included *BRAF* V600E (2.32%) and *KRAS* G12D (18.60%). Variants with potential clinical significance included several *KRAS* and an *NRAS* mutation, while variants of unknown significance included *KRAS* E49K and *NRAS* R102Q. One variant was novel: *NRAS* R102Q, and two were rare: *KRAS* E49K and G138E. We further extended the CancerVar prediction capability by adding new pathogenicity prediction tools. Molecular docking demonstrated that Encorafenib inhibits the V600E variant BRAF protein less effectively compared to its wild-type counterpart.

**Conclusion:**

Overall, this study highlights the importance of comprehensive molecular screening and bioinformatics in understanding the mutational landscape of CRC in the Saudi population, ultimately improving targeted drug treatments.

## 1 Introduction

Colorectal cancer (CRC) ranks as the third most prevalent cancer around the world, with estimations of approximately 1.9 million new cases and around 935,000 deaths in the year 2022 (Bray et al., 2024). Further estimations suggest an increase to 3.2 million new cases by the year 2040 (Morgan et al., 2023). Even as most of the cases occur among those older than 50 years, there is a significant rise in the rates of younger-onset colorectal cancers (Patel et al., 2022). In Saudi Arabia, CRC ranks first among males at 15.3% and third among females at 9.8% (Elwali et al., 2023). Most patients are present in advanced stages with symptoms of altered bowel habits, abdominal pain, rectal bleeding, and anemia (Dekker et al., 2019). CRC results from molecular changes in the colon or rectum, where there is the formation of a benign polyp that can take 5–15 years to advance into cancer (Huck and Bohl, 2016). The cause of this disease is multifactorial, with interactions between genes and the environment. About 70% of cases are considered sporadic, while 30% are inherited, with 5% being due to syndromes such as Lynch and familial adenomatous polyposis (Jasperson et al., 2010). The genetic basis of CRC has been posed to be consistent with that of the Fearon and Vogelstein model, which describes the progression from a benign adenoma to carcinoma (Fearon and Vogelstein, 1990). This model is thought to account for 70%–90% of CRC cases (Li et al., 2021). A less common way through which CRC arises is the microsatellite instability pathway, due to defects in DNA mismatch repair genes, accounting for 15%–20% of cases (Gupta et al., 2021). Lifestyle factors associated with the development of CRC include obesity, low physical activity, tobacco smoking, consumption of alcohol, and a diet high in processed red meat (Keum and Giovannucci, 2019). Endoscopic testing is considered the gold standard for diagnosing CRC, and surgical removal remains the main form of treatment for the disease (Montminy et al., 2020). In metastatic CRC (mCRC), genetic testing is used to personalize treatment protocols based on actionable somatic mutations on specific genes, namely, *BRAF*, *KRAS*, and *NRAS*, among others (Van Cutsem et al., 2016; Biller and Schrag, 2021). For patients who are not positively testing for such mutations, chemotherapy is administered with combined EGFR inhibitors. The *KRAS* and *NRAS* mutation-positive patients are treated with chemotherapy only (FOLFOX or FOLFIRI protocols) (Van Cutsem et al., 2016). Patients with the *BRAF* V600E mutation slightly benefit from chemotherapy in combination with VEGF inhibitors or a mixture of *BRAF* and EGFR inhibitors (Santarpia et al., 2012). The determination of somatic mutations in actionable genes (such as *BRAF*, *KRAS*, and *NRAS*) is vital with regards to prognosis and in offering personalized therapies. *BRAF*, *KRAS*, and *NRAS* encode core proteins in the RAS-RAF-MEK-MAPK pathway, which, if altered, can induce aberrant proliferation through dysregulating apoptosis and cell cycle progression from G1 to S phase (Seruca et al., 2009). Importantly, some detection of rare or novel variants in these actionable genes may help in the identification of new biomarkers for better therapeutic outcomes (Mikolajcik et al., 2021). In Saudi Arabia, a retrospective study from two hospitals reported a somatic mutational rate of 2.5% in the *BRAF* gene and 28.6% in *KRAS* (Siraj et al., 2014). Moreover, a similar study from another hospital revealed a 2.4% rate in *BRAF* and 30.1% in *KRAS* (Beg et al., 2015). Furthermore, a study for patients from the Gulf countries who were treated at two hospitals in the United States showed a mutational rate of 4% in *BRAF*, 44% in *KRAS*, and 4% in *NRAS* (Al-Shamsi et al., 2016). A more recent study from one Saudi hospital has shown slightly different findings for *BRAF* (0.4%), *KRAS* (49.6%), and *NRAS* (2%) (Alharbi et al., 2021). Additionally, a recent review has combined mutational rates from several other Saudi hospitals, showing 0.4%–2.5% rate for *BRAF*, 28.6%–56% in *KRAS*, and 2%–2.2% in NRAS (Alfahed, 2023) Our understanding of the mutational landscape of CRC in the Saudi population is lacking, and the percentage of common somatic mutations reported from Saudi hospitals is sparse. Moreover, diagnostic labs may sometimes ignore the rare mutations in CRC tumors, which could affect treatment outcomes. Overall, the genetic characterization of CRC is largely incomplete due to its complex nature. We analyzed CRC samples for somatic mutations in the *BRAF*, *KRAS*, and *NRAS* genes for the TruSight Tumor 15 gene panel (TST 15). The 15 genes covered by the TST 15 panel include *AKT1*, *BRAF*, *EGFR*, *ERBB2*, *FOXL2*, *GNA11*, *GNAQ*, *KIT*, *KRAS*, *MET*, *NRAS*, *PDGFRA*, *PIK3CA*, *RET*, and *TP53* genes, which are frequently altered in solid tumors (Kopetz et al., 2015; Yan et al., 2015; Van Cutsem et al., 2016; Voskoboynik et al., 2016). Therefore, to understand the pathogenicity and clinical relevance of both known and novel variants, an extensive computational analysis was performed. We have combined raw DNA sequence analysis, variant clinical interpretation, pathogenicity prediction, functional domain mapping, secondary structure and stability analyses, and 3D structure superimposition. In addition, molecular docking was performed on Encorafenib against both the BRAF V600E variant protein and its wild-type counterpart. This integrated approach aims to provide insight not only into the novel biomarkers but also to extend the knowledge of the mutational spectrum of CRC in the Saudi population, helping to further refine precision oncology interventions.

## 2 Materials and methods

### 2.1 Patient selection and data collection

This study utilized CRC patient data from King Abdulaziz University Hospital (KAUH), Jeddah, Saudi Arabia. The ethical approval was obtained from the King Abdulaziz University College of Medicine Ethical Committee (No. 487-22). All data, including raw DNA sequences, were collected from the Molecular Diagnostics Laboratory at KAUH over 4 years (2018–2021). Molecular screening was conducted using the TST 15 panel on the MiSeqDx next-generation sequencing (NGS) platform (Illumina, San Diego, CA, United States).

### 2.2 Inclusion/exclusion criteria

The study included ∼140 mm^2^ colorectal tissue samples with above 30% tumor content from male and female patients. Surgeons at KAUH performed biopsies on those patients, and the KAUH Pathology Department examined these samples histopathologically. The histopathological types considered were adenoma, adenocarcinoma, cystic, mucinous, serous neoplasms, neoplasms not otherwise specified (NOS), squamous cell neoplasms, ductal and lobular neoplasms, complex epithelial neoplasms, mature B-cell lymphomas, epithelial neoplasms NOS, lipomatous neoplasms, and chronic colon inflammation. Samples that originated from tumors outside the colon or rectum or derived from whole blood were excluded. The collected data included patient demographics, clinical and histopathology diagnoses, molecular screening results, and raw DNA sequence files for the complete gene panel (*AKT1*, *BRAF*, *EGFR*, *ERBB2*, *FOXL2*, *GNA11*, *GNAQ*, *KIT*, *KRAS*, *MET*, *NRAS*, *PDGFRA*, *PIK3CA*, *RET*, *TP53*). After excluding tumors of other types of cancer, 86 samples out of 90 collected were eligible for this study. Corresponding to those samples, 49 raw DNA sequence files were available. The remaining files were missing due to patient requests.

### 2.3 Molecular screening

The TST 15 panel screens 15 genes frequently mutated in solid tumors, targeting hotspot exonic regions for single nucleotide variants (SNVs) and small insertions/deletions (Indels). The panel design is based on international guidelines by the College of American Pathologists (CAP), the International Association for the Study of Lung Cancer (IASLC), the Association for Molecular Pathology (AMP), and the American Society of Clinical Oncology (ASCO) (Andre et al., 2012; Gonzalez et al., 2013; Lindeman et al., 2013; Sepulveda et al., 2017). DNA extractions were performed from formalin-fixed paraffin-embedded (FFPE) colorectal tissue samples (∼140 mm^2^ tissue with above 30% tumor content) using the QIAamp DNA FFPE Tissue Kit. DNA libraries were prepared using the TruSight Tumor 15 MiSeqDx kit according to the manufacturer’s instructions. Sequencing was achieved by the MiSeqDx NGS system, generating 2 × 150 bp reads using a 5% variant allele frequency (VAF) detection threshold. The Local Run Manager software was used to analyze the data, and runs took about 36 h to complete. Quality control relied on the following metrics: mean depth of coverage: ∼30×, Q-score: ≥80% of bases higher than Q30. Demultiplexing, generation of FASTQ files, alignment to the hg19 reference genome, and variant calling in variant call format (VCF) were all done on the TruSight Tumor 15 Analysis Module on the MiSeqDx instrument. These VCF files then served as input in subsequent bioinformatic analysis that aimed at identifying and interpreting the somatic variants in CRC samples.

### 2.4 Annotation, filtration, and clinical interpretation of variants within CRC actionable genes

Functional annotation highly facilitates NGS data analysis. We utilized the Annotate Variation (ANNOVAR) webserver tool (wANNOVAR) to annotate the raw DNA sequence files (https://wannovar.wglab.org) (Wang et al., 2010; Chang and Wang, 2012; Yang and Wang, 2015). The input settings used in wANNOVAR were default while selecting hg19 as a reference genome. The gene panel used in this study comprises 15 genes. Only variants from 3 genes (*BRAF*, *KRAS*, *NRAS*), however, were used in our computational analysis since they hold established therapeutic relevance in CRC. Accordingly, variant data were filtered by the 3 genes of interest, coding region, and functional effect (non-synonymous). Only the most common gene transcripts (*BRAF*: ENST00000288602.6, *KRAS*: ENST00000311936.3, *NRAS*: ENST00000369535.4) were used. The resulting list of variants (Table 1) was cross-checked with molecular screening results reported by the laboratory. Missing SNP ID numbers were manually retrieved from the National Center for Bioinformatics database (NCBI) (http://www.ncbi.nlm.nih.gov). To interpret variants based on clinical significance, we have used the 2017 four-tiered system by the Association for Molecular Pathology (AMP), the College of American Pathologists (CAP), and the American Society for Clinical Oncology (ASCO). The system uses 12 criteria for variant classification: 1) Evidence of being a therapeutic biomarker for FDA-approved or investigational drugs in professional guidelines. 2) Evidence of being diagnostic in professional guidelines or with consensus. 3) Evidence of being prognostic in professional guidelines or with consensus. 4) Mutation type: activating or loss of function, copy number variation (CNV), or gene fusion. 5) Variant allele frequency (50% or 100%). 6) Potential germline: tested as germline in normal tissue. 7) low frequency in population databases. 8) Reported as pathogenic in ClinVar or the Human Gene Mutation Database (HGMD). 9) Reported in the Catalogue of Somatic Mutations in Cancer (COSMIC), My Cancer Genome (MCG), the International Cancer Genome Consortium (ICGC), or the Cancer Genome Atlas Program (TCGA). 10) Predicted as pathogenic by pathogenicity prediction tools. 11) Association with disease or pathway. 12) Availability of convincing functional or population studies (Li et al., 2017).

**TABLE 1:** Genetic variants identified in CRC patients via TST 15 NGS molecular screening.

Serial No.	Gene	Genomic coordinates	Exon/CDS position	AA position	Ref/Alt allele	SNP reference ID	Frequency/Percentage	AMP/ASCO/CAP interpretation	MAF (ESP6500,1000g gnomAD, ExaC)
1	*BRAF*	chr7:140453135	14/c.T1799A	V600E	A/T	rs113488021	2/2.32%	Tier I - strong clinical significance	A/A/3.98E-6/1.65E-5
2	*KRAS*	chr12:25398284	2/c.G35C	G12A	C/G	rs121913529	3/3.48%	Tier II - potential clinical significance	A/A/A/A
3	*KRAS*	chr12:25398284	2/c.G35A	G12D	C/T	rs121913529	16/18.60%	Tier I - strong clinical significance	A/A/4.011E-5/1.976E-5
4	*KRAS*	chr12:25398285	2/c.G34C	G12R	C/G	rs121913530	1/1.16%	Tier II - potential clinical significance	A/A/A/A
5	*KRAS*	chr12:25398285	2/c.G34A	G12S	C/T	rs121913530	1/1.16%	Tier II - potential clinical significance	A/A/A/A
6	*KRAS*	chr12:25398284	2/c.G35T	G12V	C/A	rs121913529	5/5.81%	Tier II - potential clinical significance	A/A/A/A
7	*KRAS*	chr12:25398281	2/c.G38A	G13D	C/T	rs112445441	3/3.48%	Tier II - potential clinical significance	A/A/A/A
8	*KRAS*	chr12:25380313	3/c.G145A	E49K	C/T	rs2141510396	1/1.16%	Tier III - variant of unknown significance	A/A/A/A
9	*KRAS*	chr12:25380275	3/c.A183T	Q61H	T/A	rs17851045	1/1.16%	Tier II - potential clinical significance	A/A/A/A
10	*KRAS*	chr12:25378647	4/c.A351T	K117N	T/A	rs770248150	2/2.32%	Tier II - potential clinical significance	A/A/A/A
11	*KRAS*	chr12:25378585	4/c.G413A	G138E	C/T	rs754870563	3/3.48%	Tier II - potential clinical significance	A/A/3.981E-6/8.242E-6
12	*KRAS*	chr12:25378562	4/c.G436A	A146T	C/T	rs121913527	2/2.32%	Tier II - potential clinical significance	A/A/A/A
13	*NRAS*	chr1:115256530	3/c.C181A	Q61K	G/T	rs121913254	1/1.16%	Tier II - potential clinical significance	A/A/A/A
14	*NRAS*	chr1:115252335	4/c.G305A	R102Q	C/T	rs1247510820	1/1.16%	Tier III - variant of unknown significance	A/A/A/A

CDS, Coding Sequence; AA, Amino acid position; Ref/Alt Allele, Reference and alternative alleles; SNP Reference ID, Single Nucleotide Polymorphism Cluster ID; AMP/ASCO/CAP Interpretation, Association for Molecular Pathology/American Society for Clinical Oncology/College of American Pathologists standard clinical interpretation; MAF, Minor Allele Frequency; A, Absent in database; ESP6500, Exome Sequencing Project v.6500 database; 1000g, 1000 genome database; gnomAD, Genome Aggregation Database; ExaC, Exome Aggregation Consortium Database.

To facilitate variant interpretation, we used the CancerVar (Cancer Variant Interpretation) webserver (https://cancervar.wglab.org). CancerVar is a deep learning rule-based scoring software containing clinical records for 13 million variants from 1911 cancer census genes (Li et al., 2022). The input parameters specified were cancer type: colorectal cancer, genome build: hg19, and the amino acid position. The resulting interpretation along with the output for population frequencies were recorded (Table 1).

Pathogenicity prediction tools are helpful means for estimating the ability of genetic variants to cause disease. To classify variants as pathogenic or benign CancerVar uses seven tools, which are FATHMM (Shihab et al., 2013), GERP++RS (Davydov et al., 2010), MetaLR (Chen et al., 2023), MetaSVM (Kim et al., 2017), Mutation Assessor (Reva et al., 2011), Polyphen2_HDIV (Adzhubei et al., 2013), and SIFT (Ng and Henikoff, 2003). We have selected a different set of tools [BayesDel_addAF (Feng, 2017), CADD_Phred (Rentzsch et al., 2019), DANN (Quang et al., 2015), FATHMM-MKL (Shihab et al., 2015), MPC (Samocha et al., 2017), REVEL (Ioannidis et al., 2016), PrimateAI (Sundaram et al., 2018)] to improve pathogenicity predictions of identified variants. The reason behind selecting this tool set is to increase the diversity of principles used in prediction while offering alternative tools with more advanced algorithms. We have chosen FATHMM-MKL, for example, since it is an updated version of FATHMM, which uses multiple kernel learning to capture different types of data. Selecting CADD_phred and REVEL was based on their ability to combine the results of several tools into one comprehensive prediction. DANN and PrimateAI were selected for their advanced deep neural network algorithms that enable them to analyze complex patterns. We chose CADD_Phred and BayesDel_addAF since they incorporate diverse data types, including functional annotations, evolutionary conservation, and allele frequencies. MPC was chosen since it is tailored for missense mutations to increase prediction precision. PrimateAI is built with specific primate data, which enhances prediction for human variants; thus, it was added to our set. Therefore, this combination was consolidated to provide a more holistic approach with an expectation for increased overall prediction accuracy. We compared the performance of these tools with CancerVar by testing truly pathogenic (14 identified variants—Table 1) and truly benign (≥1% minor allele frequency) datasets. Variant Effect Predictor (VEP) was used to generate the prediction scores (McLaren et al., 2016) by inputting genomic coordinates for pathogenic variants and SNP IDs for benign variants. CancerVar tools prediction scores were obtained through its webserver, which were normalized to match the selected tools’ cutoff points. The scores for both sets (14 tools) were plotted in a receiver operating characteristic curve (ROC) using the EasyROC webtool (http://www.biosoft.erciyes.edu.tr/app/easyROC).

### 2.5 Genotype-protein-phenotype analysis of variants in CRC actionable genes

Gene-protein-phenotype characterization of cancer genes offers insights for assigning new predictive or prognostic biomarkers or designing targeted drugs. Multiple bioinformatics analyses were performed for variants found in samples that tested positive for at least one clinically actionable CRC gene (*BRAF*, *KRAS*, or *NRAS*) (Sepulveda et al., 2017). Other concomitant variants found only upon examining raw DNA sequences were also included in the analyses. Functional domain mapping, pathogenicity predictions, secondary structure analysis, thermodynamic stability analysis, 3D structure superimposition, and molecular docking of BRAF wild-type and mutant proteins to Encorafenib (a BRAF inhibitor) were performed. All actionable gene variants, including concomitant variants, were missense variants in the coding region. To identify novel or rare variants, a thorough search was conducted in major population databases [1000 genomes, Avon Longitudinal Study of Parents and Children (ALSPAC), The Exome Aggregation Consortium (ExAC), The Genome Aggregation Database (GnomAD), National Center for Biotechnology Information Allele Frequency Aggregator (NCBI ALFA), NHLBI Exome Sequencing Project (ESP6500), TWINSUK, UK10K], and cancer-specific databases (COSMIC, MCG, and TCGA).

#### 2.5.1 Functional domain mapping

All of the identified missense variants in *BRAF*, *KRAS*, and *NRAS* genes were mapped to exons, domains, and subdomains based on the primary sequence of amino acids from literature consensus. The chromosome mapping location, mRNA transcripts, and corresponding exon numbers were collected from the Ensembl Database (https://www.ensembl.org/index.html).

#### 2.5.2 Secondary structure and stability analyses

Secondary structure analysis illustrates differences in secondary structure elements caused by variations in amino acids. Amino acid sequences of wild-type proteins (BRAF, KRAS, and NRAS) were manually substituted in a text file with each variation, and sequences were analyzed using the NetSurfP 3.0 webtool (https://www.dtu.biolib.com/NetSurfP-3) to generate graphical images. Those images were visually compared to see whether any changes occurred in α-helices, ß-pleated sheets, or loops between wild-type and variant secondary structures.

Thermodynamic stability analysis estimates the mutational effect on protein stability. *BRAF*, *KRAS*, and *NRAS* missense variants were analyzed using the DUET webserver, predicting the effect of SNVs on protein stability in the form of ΔΔG (kcal/mol). DUET integrates mCSM and SDM estimations to predict a combined score (Pires et al., 2014).

#### 2.5.3 3D structure mapping and superimposition

Variant tertiary structure generation and alignment to the respective wild-type model were achieved using the homology modeling method. Obtained from the Protein Databank (PDB) (https://www.rcsb.org), folded human 3D structures of BRAF (PDB: 6UAN), KRAS (PDB: 7KYZ), and NRAS (PDB: 6ZIO) were used as templates to generate the wild-type forms of the proteins. Wild-type 3D structures were generated using SWISS-MODEL webserver (https://www.swissmodel.expasy.org) (Waterhouse et al., 2018), except for BRAF, which was achieved throughthe I-Tasser webserver (https://www.zhanggroup.org) (Zhang, 2008; Roy et al., 2010; Yang et al., 2015), since it needed input from multiple structures to generate the full form. The mutant forms were made by the DUET webserver (Pires et al., 2014). Deviations were estimated by superimposing mutant residues on wild-type counterparts on YASARA software (https://www.yasara.org) (Land and Humble, 2018), with root mean square deviation (RMSD) values generated as outputs. The PyMol2 software (PyMOL Molecular Graphics System Ver. 2.6.0, Schrödinger, LLC) was used to visualize the aligned protein structures along with affected amino acids.

#### 2.5.4 Computational binding of BRAF with Encorafenib

According to American guidelines, Encorafenib (a kinase inhibitor) in addition to Cetuximab (an EGFR inhibitor) can be used as a second line of treatment for mCRC patients with the *BRAF* V600E mutation (Morris et al., 2023). Moreover, the maximum inhibitory concentration (IC50) of Encorafenib *in vitro* is known to be similar in wild-type and V600E mutant BRAF proteins (Koelblinger et al., 2018). To gain more insight on Encorafenib binding behavior, molecular docking assays were performed to compare the binding affinities of Encorafenib with wild-type and V600E mutant BRAF proteins. AutoDock Vina algorithm of the SWISS-DOCK webserver (https://www.swissdock.ch) was utilized to assess two assays: one for the drug-wild-type and the other for the drug-variant (Eberhardt et al., 2021; Bugnon et al., 2024). For the docking setup, the ligand was obtained from the ZINC database in MOL2 format (zinc.docking.org) (catalogue no.: ZINC68249103) and uploaded into the SWISS-DOCK server. The molecule sketcher option was used to prepare the uploaded ligand. The previously generated wildtype and variant models (methods 2.5.3) were used in PDBQ format to prepare target molecules. For each model, specific amino acid coordinates (inhibitor binding residues) were entered in the search box center of grid box settings (Martinez Fiesco et al., 2022). The search box size and sample exhaustivity options were left on default. The drug-protein complexes with the lowest binding energies were chosen, and PyMol2 was used to record hydrogen bonds and interacting residues and visualize the results.

## 3 Results

### 3.1 Data collection

The sample’s data collected for a 4-year period (2018–2021) comprise information on clinical diagnosis, demographics, histopathology diagnosis, and molecular screening results for the 3 CRC actionable genes (*BRAF*, *KRAS*, *NRAS*). Some raw DNA sequence files for the full gene panel were also collected. Upon curation and filtration, the data collection process has yielded a total of 86 CRC sample data, with 49 raw DNA sequence files corresponding to some of those samples. Among the 86 samples, 41 harbored somatic mutations in CRC actionable genes, and 45 were negative for those genes. All 86 samples’s data displayed diverse clinical presentations. These include colon or rectal polyps, transverse, descending, sigmoid, rectosigmoid, non-specified colon, or rectal cancers, and chronic colon inflammation. The demographic data shows a higher number of samples for males (n = 51, 59.30%) compared to females (n = 35, 40.70%). The majority of these samples belonged to patients between the ages of 50 and 59 years old (n = 29, 33.72%), followed by patients between 60 and 69 years old (n = 22, 25.58%), 70 and 79 years old (n = 18, 20.93%), 40 and 49 years old (n = 12, 13.95%), and 30 and 39 (n = 3, 3.49%). Other remaining samples were for a patient between <30 years old (n = 1, 1.16%) and a patient >80 years old (n = 1, 1.16%). The histopathology diagnosis data of 86 patients shows a diverse range of histologic types, with each type present in small numbers. Therefore, to simplify observation for these data, each patient tissue diagnosis was broadly grouped into either adenoma (precursor lesion), adenocarcinoma (malignant form), or chronic colon inflammation. The majority of sample tissues were diagnosed with adenocarcinoma (n = 71, 82.5%), followed by adenoma (n = 9, 10.5%), and chronic colon inflammation (n = 6, 7%).

### 3.2 Molecular screening and variant interpretation

The 14 detected variants in 3 actionable genes among 86 samples were as follows: *BRAF*, n = 1 (1.16%); *KRAS*, n = 11 (12.79%); and *NRAS*, n = 2 (2.32%). The single *BRAF* variant was V600E. Among the 11 *KRAS* variants, 5 were in codon 12 (G12A, G12D, G12R, G12S, G12V) and 1 in codon 13 (G13D). Two variants of *KRAS*, E49K and G138E, were a concomitant pair from a single patient. The other variants of *KRAS* included codon 61 Q61H, codon 117 K117N, and codon 146 A146T. The 2 *NRAS* variants included a codon 61 mutation (Q61K) and a rare mutation at codon 102 (R102Q). The 14 variant identifications were based on molecular screening records and 49 corresponding raw DNA sequence files. All variants found in the molecular screening records were present in raw DNA sequence files, except for *NRAS* R102Q, which was only identified in those files. Moreover, all 14 variants were found above the 5% VAF threshold except for *NRAS* R102Q, which was detected at 1.4%. It was included, however, in subsequent computational analyses as it may hold potential clinical relevance upon further investigation. Furthermore, the *NRAS* R102Q variant was found concomitantly with another variant (*KRAS* G12D) in the same patient. The most common variant detected was *KRAS* G12D (n = 16, 18.60%). According to CancerVar interpretation, *BRAF* V600E and *KRAS* G12D were classified as Tier 1 (strong clinical significance), while *KRAS* E49K and *NRAS* R102Q were Tier III (unknown clinical significance). *KRAS* E49K is a rare variant, and *NRAS* R102Q is a novel variant. According to the COSMIC database, *KRAS* E49K is only reported twice from large intestine samples and once from a lung sample (COSV55695731), while the *NRAS* R102Q is reported only once from a thyroid sample (COSV106063871), but never from large intestines. No clinical significance data, however, for these variants is available or reported in other databases. The remaining variants were Tier II (potential clinical significance): *KRAS* G12A, G12R, G12S, G12V, G13D, Q61H, K117N, G138E, A146T, and *NRAS* Q61K. *KRAS* G138E is a rare variant, only reported in COSMIC twice from large intestine samples and once from an endometrium sample (COSV55967074). Moreover, this variant was found in the ExAC database at a very low allele frequency (0.00000824%). Furthermore, all 14 variants are mostly absent or present at very low allele frequencies in population databases (Table 1).

### 3.3 Functional domain mapping

**FIGURE 1:**
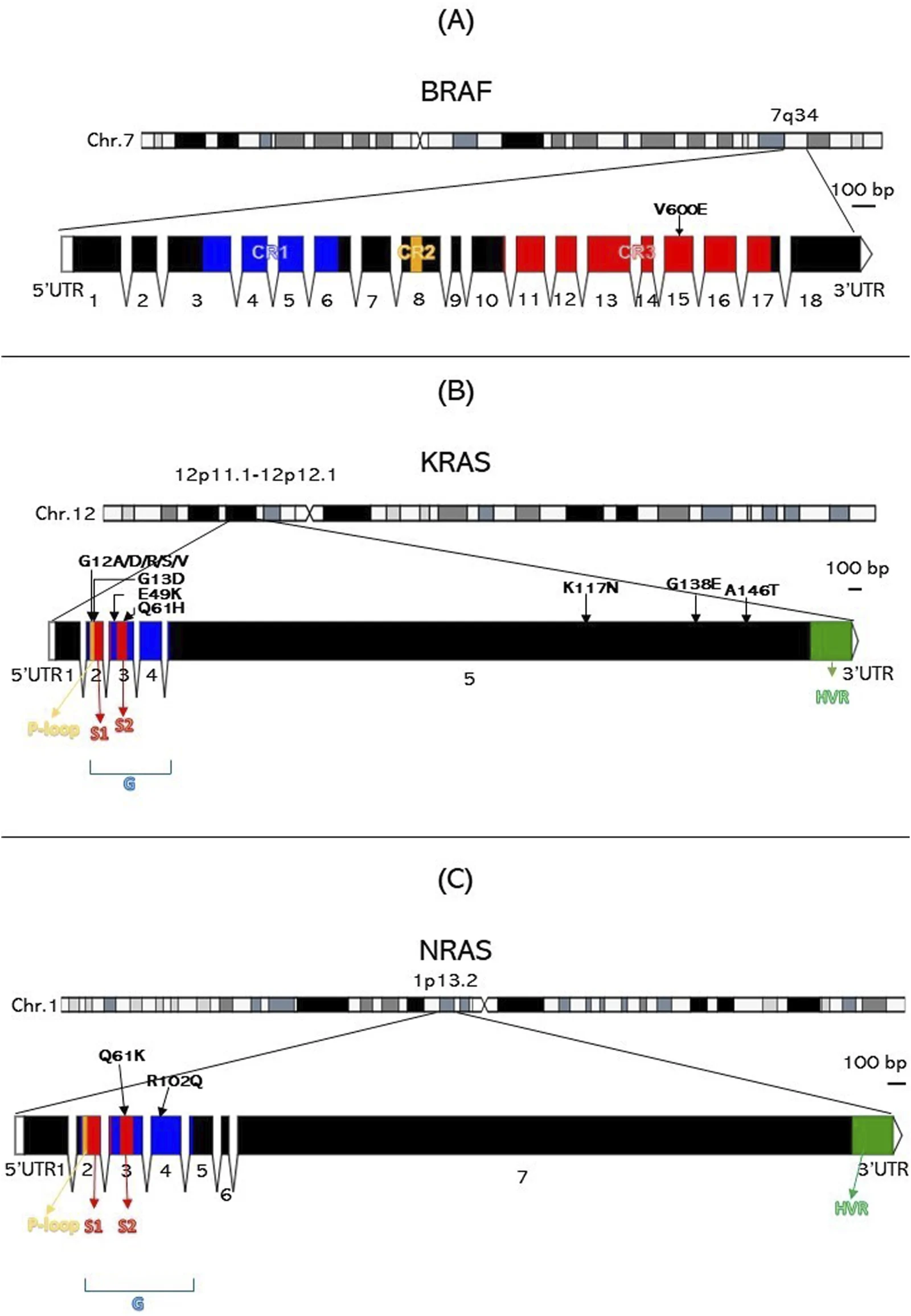
Genomic architecture and variant mapping for *BRAF*, *KRAS*, and *NRAS*. This figure is divided into three parts: **(A)** for *BRAF*, **(B)** for *KRAS*, and **(C)** for *NRAS*, each illustrating the chromosome location, exon structure, functional domains, and the specific locations of the 14 variants identified in the study. HVR, Hypervariable region. Numbers indicate exons.

The single variant of *BRAF* (V600E) is situated at exon 15 in the BRAF kinase binding domain’s CR3 lobe (residues ∼442–724) (Cope et al., 2018) (Figure 1A). *KRAS* codon 12 and 13 variants (G12A, G12D, G12R, G12S, G12V, and G13D) are located at the second exon in the catalytic (G) domain’s embedded P-loop region (residues ∼10-16 and 56–59). *KRAS* E49K and Q61H are in exon 3, with Q61H within the S2 domain (residues ∼58–76). *KRAS* K117N, G138E, and A146T variants are located in exon 5 near the hypervariable region (HVR) (residues ∼167–188) (Figure 1B). The *NRAS* Q61K and R102Q variants are within the G domain (residues ∼1–165), with Q61K in exon 3 and the S2 domain, and R102Q in exon 4, not associated with other domains (Johnson et al., 2017) (Figure 1C).

### 3.4 Pathogenicity predictions

Our pathogenicity prediction analysis was performed on the 14 variants using two sets of tools: 7 CancerVar tools and our 7 study-selected tools. The output was evaluated based on the 0.5 threshold (>0.5 = pathogenic and <0.5 = benign). For each set, we consider a variant to be pathogenic if >3 tools scored above the threshold, while we consider the variant to be benign if ≤3 tools scored above the threshold. Between the 7 CancerVar tools and our 7 selected tools, with better prediction algorithms, there was great variability in pathogenicity predictions. CancerVar tools predicted three variants—*KRAS* G12S, E49K, and *NRAS* R102Q—to be benign (42.85%, 28.57%, and 28.57, respectively), but our tools identified them as pathogenic (100%, 85.71%, and 100%, respectively).

*KRAS* G12S, predicted benign by 3 CancerVar tools (SIFT, GERP++RS, and PolyPhen2_HDIV), and other 4 tools considered it as pathogenic. But all selected tools (100%) predicted it as pathogenic. Similarly, *KRAS* E49K was predicted as benign by CancerVar (2 tools: SIFT, GERP++RS) but pathogenic by 6 tools (85.71%) in our study (MPC, BayesDel_addAF, REVEL, FATHMM-MKL, PrimateAI, DANN). Lastly, *NRAS* R102Q, predicted benign by CancerVar (2 tools: SIFT, GERP++RS), was considered pathogenic by all 7 tools (100%) in our study (MPC, BayesDel_addAF, CADD_Phred, REVEL, FATHMM-MKL, PrimateAI, DANN). All other variants (*BRAF* V600E, *KRAS* G12A, G12D, G12R, G12V, G13D, Q61H, K117N, G138E, A146T, *NRAS* Q61K) were predicted as pathogenic by both sets of tools (Tables 2, 3).

**TABLE 2:** Pathogenicity prediction analysis: comparison of pathogenicity prediction scores generated by 2 different sets of tools (CancerVar tools versus study selected tools) for 14 missense variants identified in CRC patients.

Serial No.	Gene	AA position	CancerVar pathogenicity prediction tools							Study selected pathogenicity prediction tools						
			SIFT	GERP++RS	MetaSVM	PolyPhen2 HDIV	MetaLR	FATHMM	Mutation Assessor	MPC	BayesDel addAF	CADD phred	REVEL	FATHMM-MKL	PrimateAI	DANN
1	*BRAF*	V600E	0.99	0.9375	0.192	0.971	0.259184	0.501025	0.043279381	0.98012	0.89859	0.85358	0.98378	0.83898	0.9559	0.42049
2	*KRAS*	G12A	0.7	0.941106	0.534281	0.956	0.574	0.485573	0.536671058	0.97803	0.79266	0.66338	0.95026	0.83094	0.95412	0.89353
3	*KRAS*	G12D	0.76	0.941106	0.542977	0.517	0.581	0.487634	0.643719807	0.96146	0.89791	0.55279	0.9628	0.77787	0.98933	0.86433
4	*KRAS*	G12R	0.8	0.941106	0.5301	0.802	0.557	0.486397	0.581137462	0.95402	0.88153	0.71141	0.94276	0.83094	0.98714	0.99279
5	*KRAS*	G12S	0.7	0.941106	0.0365	0.682	0.061224	0.480214	0.076706545	0.96251	0.8702	0.76569	0.93068	0.77787	0.95359	0.94547
6	*KRAS*	G12V	0.9	0.941106	0.58913	0.999	0.699	0.488046	0.631642512	0.96812	0.89074	0.71712	0.97587	0.82057	0.96271	0.87839
7	*KRAS*	G13D	0.68	0.941106	0.593478	0.803	0.69	0.467436	0.657992973	0.97311	0.91571	0.55802	0.94691	0.77787	0.98766	0.91714
8	*KRAS*	E49K	0.71	0.951923	0.133	0.083673	0.2	0.477329	0.099929627	0.94702	0.74091	0.49447	0.75074	0.8248	0.98822	0.92925
9	*KRAS*	Q61H	0.92	0.951923	0.542475	0.080612	0.613	0.510595	0.636583224	0.92349	0.80121	0.52932	0.86636	0.56224	0.9584	0.82054
10	*KRAS*	K117N	0.88	0.049545	0.648997	1	0.948	0.527683	0.836407554	0.43041	0.84787	0.63498	0.92004	0.54318	0.98302	0.92576
11	*KRAS*	G138E	0.99	0.921875	0.573579	0.967	0.687	0.469085	0.524044796	0.38983	0.86851	0.71935	0.86986	0.8027	0.99825	0.83409
12	*KRAS*	A146T	0.91	0.921875	0.66689	1	0.89	0.531442	0.720575318	0.35994	0.92729	0.79742	0.97549	0.97329	0.93982	0.96049
13	*NRAS*	Q61K	0.91	0.86899	0.615719	0.948	0.742	0.507861	0.696969697	0.80574	0.90961	0.75616	0.95026	0.9545	0.94521	0.68618
14	*NRAS*	R102Q	0.62	0.91226	0.16025	0.378571	0.239796	0.4831	0.066502463	0.76201	0.73138	0.5419	0.82968	0.73196	0.90151	0.94815

AA position, Change in amino acid, Cutoff values: variants are predicted pathogenic if score is >0.5 (red color) and benign if <0.5 (green color).

**TABLE 3:** Overall pathogenicity predictions of 14 missense variants identified in CRC Patients.

Serial No.	Gene	AA position	SNP Ref. ID	AMP/ASCO/CAP interpretation	CancerVar pathogenicity prediction	Selected tools pathogenicity prediction
1	*BRAF*	V600E^a^	rs113488022	Tier I - strong clinical significance	Pathogenic	Pathogenic
2	*KRAS*	G12A^a^	rs121913529	Tier II - potential clinical significance	Pathogenic	Pathogenic
3	*KRAS*	G12D^a^	rs121913529	Tier I - strong clinical significance	Pathogenic	Pathogenic
4	*KRAS*	G12R^a^	rs121913530	Tier II - potential clinical significance	Pathogenic	Pathogenic
5	*KRAS*	G12S^a^	rs121913530	Tier II - potential clinical significance	Benign	Pathogenic
6	*KRAS*	G12V^a^	rs121913529	Tier II - potential clinical significance	Pathogenic	Pathogenic
7	*KRAS*	G13D^a^	rs112445441	Tier II - potential clinical significance	Pathogenic	Pathogenic
8	*KRAS*	E49K^b^	rs2141510396	Tier III - variant of unknown significance	Benign	Pathogenic
9	*KRAS*	Q61H^a^	rs17851045	Tier II - potential clinical significance	Pathogenic	Pathogenic
10	*KRAS*	K117N^a^	rs770248150	Tier II - potential clinical significance	Pathogenic	Pathogenic
11	*KRAS*	G138E^b^	rs754870563	Tier II - potential clinical significance	Pathogenic	Pathogenic
12	*KRAS*	A146T^a^	rs121913527	Tier II - potential clinical significance	Pathogenic	Pathogenic
13	*NRAS*	Q61K^a^	rs121913254	Tier II - potential clinical significance	Pathogenic	Pathogenic
14	*NRAS*	R102Q^c^	rs1247510820	Tier III - variant of unknown significance	Benign	Pathogenic

Predictions of both CancerVar and study selected tools are shown for comparison.

AA, amino acid position; SNP Reference ID, Single Nucleotide Polymorphism Cluster ID; AMP/ASCO/CAP Interpretation, Association for Molecular Pathology/American Society for Clinical Oncology/College of American Pathologists standard clinical interpretation.

^a^ Actionable variants identified through initial standard screening.

^b^ Non-actionable variants identified through initial standard screening.

^c^ Non-actionable variants identified through raw DNA sequence analysis.

**FIGURE 2:**
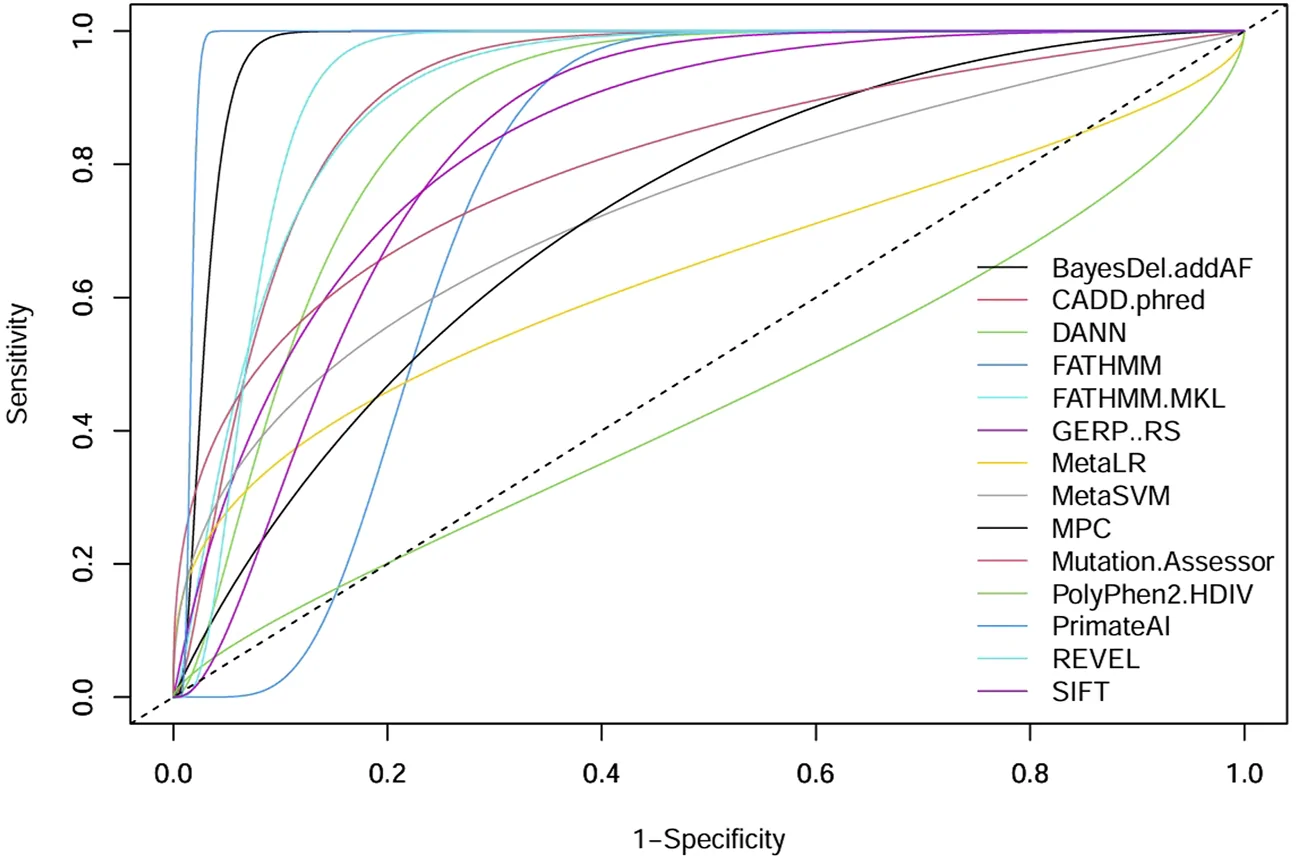
Comparative performance of 14 pathogenicity prediction tools. Two different sets of pathogenicity prediction tools, each containing 7 tools are compared to demonstrate their ability to detect pathogenic from benign variants. One set (SIFT, GERP++RS, MetaSVM, PolyPhen2 HDIV, MetaLR, FATHMM, and Mutation Assessor) are used by the CancerVar somatic variant interpretation tool, and the other set (MPC, BayesDel addAF, CADD phred, REVEL, FATHMM-MKL, PrimateAI, and DANN) are selected by our study to be used as a better predictor for pathogenicity.

ROC curves can be a good indicator of the performance of different pathogenicity prediction tools. By plotting the true positive and true negative rates, area under the curve (AUC) can be calculated. A perfect performance for a given tool is 1, and unreliable tools score 0.5 or below. Upon comparison in ROC analysis, CancerVar’s 7 tools (SIFT, GERP++RS, MetaSVM, PolyPhen2_HDIV, MetaLR, FATHMM, MutationAssessor) and our 7 selected tools (MPC, BayesDel_addAF, CADD_Phred, REVEL, FATHMM-MKL, PrimateAI, DANN) displayed wide variations in terms of performance (Figure 2). Five of the selected tools (PrimateAI, BayesDel_addAF, REVEL, FATHMM-MKL, and CADD_Phred) demonstrated outstanding performance, with AUC values above 0.90 (0.98292, 0.96854, 0.92479, 0.90957, and 0.90922, respectively). Additionally, DANN showed a slightly lower AUC value (0.87218), which also indicates a high performance. MPC was the least performing among this group with an AUC of 0.72212. Therefore, the top six performing prediction tools in this analysis (PrimateAI, BayesDel_addAF, REVEL, FATHMM-MKL, CADD_Phred, and DANN) were among our study-selected tools. CancerVar tools showed lower AUC values for GERP++RS, SIFT, and Mutation Assessor (0.84312, 0.83093, and 0.80124, respectively) compared to the top 6 performing selected tools. FATHMM and MetaSVM had even lower AUC values (0.77022, 0.73343, respectively) but were still better than the selected MPC tool. MetaLR and PolyPhen2_HDIV were the least performing among all other tools, with AUC values below 0.70 (0.63529 and 0.44089, respectively). All results were statistically significant (*p*-value <0.05) (Table 4). Overall, our selected tools, with an average AUC of 0.89848, significantly improved prediction ability by approximately 24.4% compared to CancerVar tools, which had an average AUC of 0.72216.3.5.

**TABLE 4:** Performance evaluation of 14 pathogenicity prediction tools.

Pathogenicity prediction tools		AUC	SE AUC	Lower limit	Upper limit	Z-score	*p*-value
CancerVar Tools	SIFT	0.83093	0.08408	0.66614	0.99572	3.93604	0.000080*
	GERP++RS	0.84312	0.07597	0.69422	0.99202	4.51648	0.000010*
	MetaSVM	0.73343	0.09717	0.54298	0.92388	2.40224	0.016300*
	PolyPhen2_HDIV	0.44089	0.11113	0.22307	0.65871	−0.53185	0.594830
	MetaLR	0.63529	0.10843	0.42277	0.8478	1.24773	0.212130
	FATHMM	0.77022	0.09454	0.58492	0.95552	2.85812	0.004260*
	MutationAssessor	0.80124	0.08556	0.63355	0.96892	3.52092	0.000430*
Study Selected Tools	MPC	0.72212	0.09669	0.5326	0.91163	2.29716	0.021610*
	BayesDel_addAF	0.96854	0.13875	0.69659	1	3.37677	0.000730*
	CADD_phred	0.90922	0.07915	0.75408	1	5.16998	0*
	REVEL	0.92479	0.10432	0.72034	1	4.07216	0.000050*
	FATHMM-MKL	0.90957	0.07578	0.76104	1	5.40443	0.000000*
	PrimateAI	0.98292	0.19461	0.60149	1	2.4815	0.013080*
	DANN	0.87218	0.07979	0.71579	1	4.66442	0*

Each tool, including CancerVar and our selected set, was tested on pathogenic and benign datasets, with results plotted on an ROC curve. This table summarizes the associated statistics.

AUC, area under curve; SE AUC, Standard error of area under curve. The color intensity illustrates the performance each prediction tool. The darker the green shade the better performing, and the lighter the less performing. * = A statistically significant difference from random classification (*p*-value <0.05).

### 3.5 Secondary structure, stability, and structural deviation analyses

Secondary structure analysis determines how amino acid changes in the protein may affect the size or shape of secondary structure elements (α-helices, ß-pleated sheets, and loops). Interestingly, the output generated by the NetSurfP 3.0 tool showed that 12 variants are falling within loop regions without notable changes in length or shape of the loops (*BRAF* V600E, *KRAS* G12A, G12D, G12R, G12S, G12V, G13D, Q61H, K117N, G138E, A146T, and *NRAS* Q61K). *KRAS* E49K variant falls within a ß-pleated sheet region, and *NRAS* R102Q within an α-helix. Both of these variants did not cause any visible change in their respective secondary structure elements (Supplementary Figure 1).

Thermodynamic stability analysis predicts the impact of mutations on protein folding. Variants making changes to protein thermodynamic stability are predicted to affect protein function. Actionable gene missense variants were analyzed using different measurements to estimate changes in energy. The DUET webtool combines the output of two different tools, mutation cutoff scanning matrix (mCSM) and site-directed mutator (SDM), to make its final prediction. DUET webserver predicted the *BRAF* V600E variant to be destabilizing the protein structure due to the negative free energy value (−2.118 kcal/mol). Seven *KRAS* variants were also shown to have a destabilizing effect: G12D (−0.323 kcal/mol), G12R (−0.044 kcal/mol), G12S (−0.274 kcal/mol), G13D (−0.461 kcal/mol), K117N (−0.455 kcal/mol), G138E (−1.28 kcal/mol), and A146T (−0.737 kcal/mol). Other 4 *KRAS* variants were found to further stabilize the protein structure due to the positive energy change: G12A (0.056 kcal/mol), G12V (0.168 kcal/mol), E49K (0.063 kcal/mol), and Q61H (0.256 kcal/mol). Both *NRAS* variants were also found to cause increased protein stability: Q61K (0.486 kcal/mol) and R102Q (0.012 kcal/mol). Compared to other analyzed variants, *BRAF* V600E was shown to be severely destabilizing the protein structure due to its large negative value, and *NRAS* R102Q to be weakly stabilizing its protein due to the small positive value (Table 5).

**TABLE 5:** RMSD and thermodynamic stability scores of 14 missense variants identified in CRC patients.

Serial No.	Gene	AA position	SNP reference ID	Residue level RMSD	mCSM stability change (kcal/mol)	SDM stability change (kcal/mol)	DUET stability change (kcal/mol)
1	*BRAF*	V600E	rs113488022	1.7216	−1.957 (Destabilizing)	−2.11 (Destabilizing)	−2.118 (Destabilizing)
2	*KRAS*	G12A	rs121913529	1.1634	−0.193 (Destabilizing)	−0.35 (Destabilizing)	0.056 (Stabilizing)
3	*KRAS*	G12D	rs121913529	1.1833	−0.554 (Destabilizing)	−0.74 (Destabilizing)	−0.323 (Destabilizing)
4	*KRAS*	G12R	rs121913530	1.1612	−0.252 (Destabilizing)	−0.25 (Destabilizing)	−0.044 (Destabilizing)
5	*KRAS*	G12S	rs121913530	1.1833	−0.437 (Destabilizing)	−1.01 (Destabilizing)	−0.274 (Destabilizing)
6	*KRAS*	G12V	rs121913529	1.1651	−0.313 (Destabilizing)	0.73 (Stabilizing)	0.168 (Stabilizing)
7	*KRAS*	G13D	rs112445441	1.5183	−0.23 (Destabilizing)	−2.92 (Destabilizing)	−0.461 (Destabilizing)
8	*KRAS*	E49K	rs2141510396	1.3381	−0.152 (Destabilizing)	−0.58 (Destabilizing)	0.063 kcal/mol (Stabilizing)
9	*KRAS*	Q61H	rs17851045	2.3574	0.01 (Stabilizing)	0.7 (Stabilizing)	0.256 (Stabilizing)
10	*KRAS*	K117N	rs770248150	2.798	−0.766 (Destabilizing)	0.44 (Stabilizing)	−0.455 (Destabilizing)
11	*KRAS*	G138E	rs754870563	1.4388	−1.074 (Destabilizing)	−2.62 (Destabilizing)	−1.28 (Destabilizing)
12	*KRAS*	A146T	rs121913527	1.5958	−0.963 (Destabilizing)	−0.57 (Destabilizing)	−0.737 (Destabilizing)
13	*NRAS*	Q61K	rs121913254	4.263	0.072 (Stabilizing)	0.25 (Stabilizing)	0.486 (Stabilizing)
14	*NRAS*	R102Q	rs1247510820	2.4139	−0.16 (Destabilizing)	−0.13 (Destabilizing)	0.012 (Stabilizing)

AA, amino acid position; Ref/Alt Allele, Reference and alternative alleles; SNP Reference ID, Single Nucleotide Polymorphism Cluster ID; RMSD, Root-Mean-Square Deviation; mCSM, mutation cutoff scanning matrix; SDM, Site-directed mutator.

**FIGURE 3:**
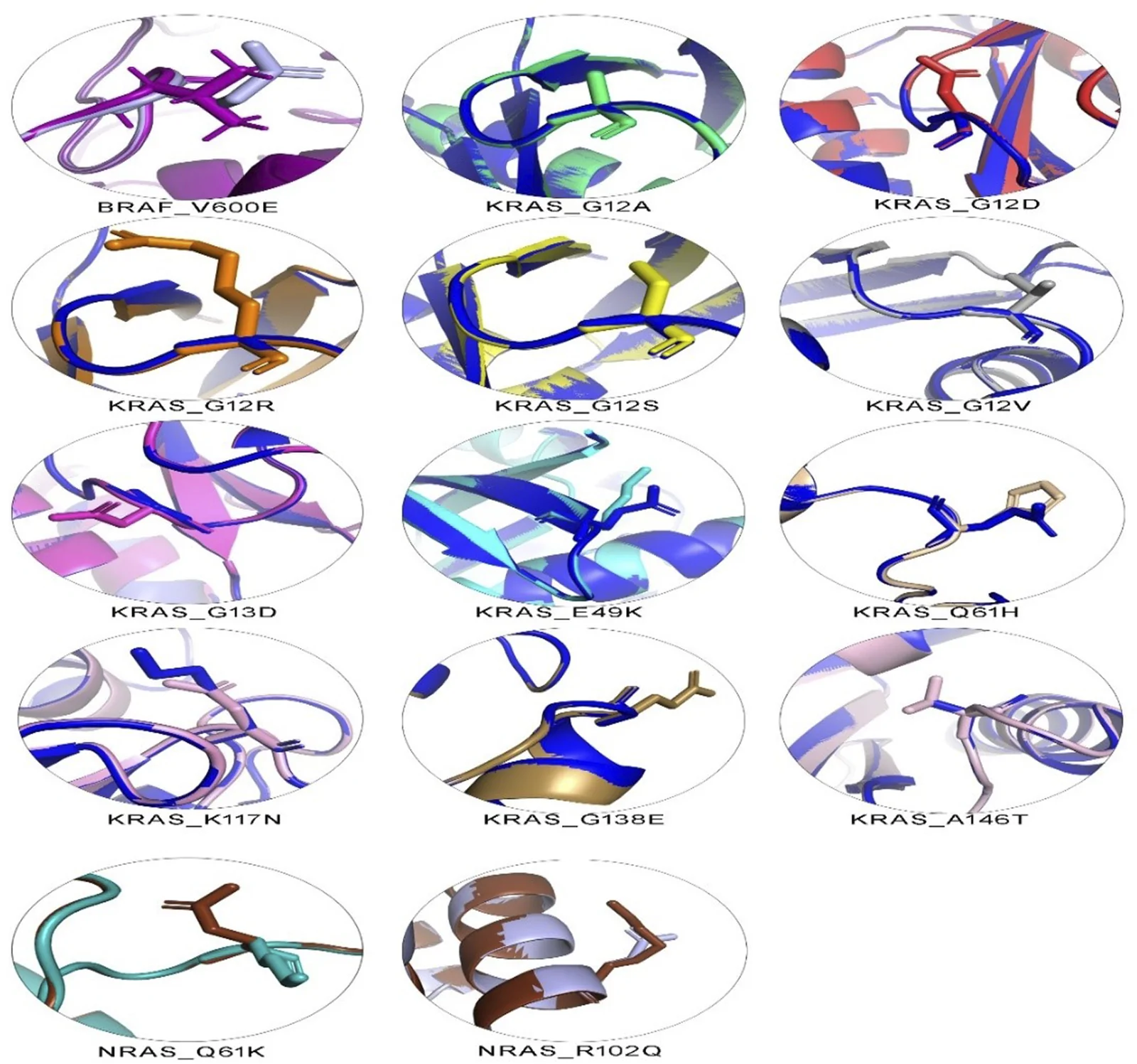
3D structural analysis of variant and wild-type BRAF, KRAS, and NRAS proteins. The figure shows 14 variant amino acid 3D structures superimposed on their wild-type counterparts. Each superimposed structure highlights the effect of its respective mutation on protein shape. Contrasting colors are shown for each variant and wild-type pair. Wild-type BRAF is shown in dark purple, wild-type KRAS in dark blue, and wild-type NRAS in dark brown.

The 3D structure superimposition of the folded mutant proteins on their wild-type counterparts was performed to estimate deviations at the amino acid level. Three variants revealed significant structural deviations (>2Å) by RMSD analysis scores. These were *KRAS* Q61H (2.3574 Å), K117N (2.798 Å), and *NRAS* R102Q (2.4139 Å). The remaining 11 variants fell below the 2Å threshold (*BRAF* V600E, *KRAS* G12A, G12D, G12R, G12S, G12V, G13D, E49K, G138E, A146T, and *NRAS* Q61K) (Table 5). Overall, the superimposed 3D structures of all 14 variants showed subtle changes at residue level (Figure 3).

### 3.6 Computational binding of BRAF with Encorafenib

**FIGURE 4:**
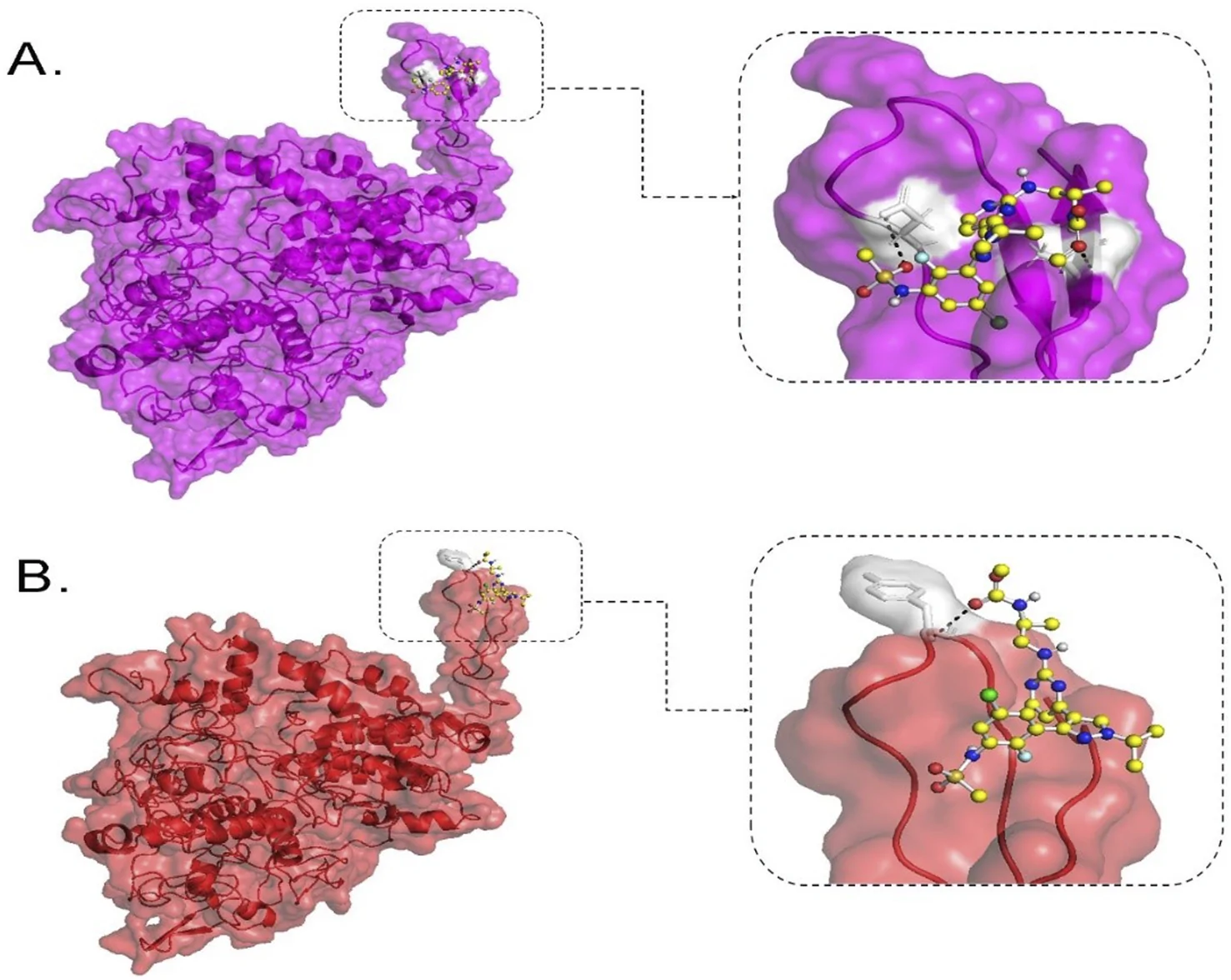
Molecular docking analysis of BRAF protein with Encorafenib. This figure shows the binding configuration of wild-type BRAF **(A)** and BRAF V600E variant protein **(B)** with Encorafenib. Encorafenib is depicted as a small molecule in a ball-and-stick illustration, with white shaded pockets indicating hydrogen bond formation sites. The dotted lines represent hydrogen bonds. In **(A)**, the wild-type BRAF protein is shown in purple on the left, with an enlarged view of the protein-ligand interaction site on the right. In **(B)**, the *BRAF* V600E variant protein is shown in red on the left, with an enlarged view of the protein-ligand interaction site on the right.

BRAF is a promising drug target for mCRC patients with the V600E mutation. Moreover, Encorafenib (a kinase inhibitor) can be used as a targeted therapy for those patients as a second- or third-line treatment option. To catch a glimpse of the efficacy of this drug, molecular docking was performed to estimate the binding affinity of this kinase inhibitor against wild-type BRAF protein and its V600E variant. The binding affinity between Encorafenib and wild-type BRAF protein was computed to −5.915 kcal/mol. It is predicted to form hydrogen bonds with two residues, D742 and A762, in the protein. However, the BRAF V600E mutant protein showed lower binding affinity with Encorafenib, with a computed value of −3.539 kcal/mol and one hydrogen bond at residue Y746 of the protein (Figure 4).

## 4 Discussion

CRC is a challenging disease. Twenty percent of patients present at a metastatic stage and 50% of localized cases progress to mCRC (Dekker et al., 2019). Despite the genetic heterogeneity of CRC tumors, targeted treatments remain limited. *BRAF*, *KRAS*, and *NRAS* express vital cascade signaling proteins that act in the RAS-RAF-MEK-MAPK pathway, ultimately influencing the cell cycle G1 checkpoint. While cell commitment to DNA synthesis is largely controlled by retinoblastoma tumor suppressor protein (Rb), mutations in these signaling proteins can promote cell cycle progression from G1 to S phase by increasing expression of cyclins and cyclin-dependent kinases that are known to nullify the effects of Rb. Such mutations in these actionable genes disrupt normal cell cycle regulation, leading to uncontrolled proliferation and carcinogenesis (Sherr, 1995; Smalley et al., 2008; Puyol et al., 2010). In this study, we collected 86 samples from KAUH hospital: 40 (46.5%) were positive and the rest were negative for mutations in these actionable genes. Fourteen variants were identified through molecular screening and raw DNA sequence analysis: 1 in *BRAF*, 11 in *KRAS*, and 2 in *NRAS*, including *NRAS* R102Q, previously unreported in CRC data. *BRAF* mutations, particularly the V600E variant, occur in 66% of melanomas and less frequently in other cancers (OMIM:164757). Mutations in *BRAF* codon 600, *KRAS* codons 12 and 13, and *NRAS* codon 61 confer resistance to anti-EGFR treatments, while *BRAF* V600E mutations indicate aggressive disease but potential benefit from VEGF inhibitors (Ciardiello et al., 2022). This variant disrupts kinase activity, increasing signaling independently of RAS, with a 138-fold rise in oncogenic activity (Oikonomou et al., 2014). It is a key biomarker in thyroid (Koh et al., 2013), melanoma (Cheng et al., 2018), and CRC (Ciardiello et al., 2022). *BRAF* mutations are present in 8%–12% of European mCRC patients and associated with poor survival (Van Cutsem et al., 2016; Tabernero et al., 2022). In this study, CancerVar identified this variant as Tier I (Table 1), consistent with ClinVar (RCV001030023.12). The frequency of *BRAF* mutations in our data is 2.32% (Table 1), aligning with Saudi data (0.4%–4%) (Siraj et al., 2014; Beg et al., 2015; Al-Shamsi et al., 2016; Alharbi et al., 2021; Alfahed, 2023).

The *BRAF* gene, located at chromosome 7q34, consists of 18 exons encoding a 766-amino acid protein (Huang et al., 2013). *BRAF* is a serine/threonine kinase that performs several functions in the RAS-RAF-MEK-MAPK pathway (Rauch et al., 2011). Under physiological conditions, growth factors activate RAS proteins at the cell membrane, which then activate RAF proteins, thus amplifying the signal. Balanced regulatory signals autoinhibit RAF as a means of turning off growth signals when not needed. *BRAF* V600E, a type 1 mutation, functions as a monomer, increasing signaling without RAS dimerization and resisting regulated inhibition (Ciombor et al., 2022). *BRAF* protein has three conserved domains: CR1, CR2, and CR3. CR1 (residues 120–280), near the N-terminus, includes cysteine-rich and RAS-binding (residues 155–227) subdomains. The cysteine-rich subdomain controls kinase activity by autoinhibition (Cutler et al., 1998; Zaman et al., 2019), while the RAS-binding domain upregulates *BRAF* at the plasma membrane (Freeman et al., 2013). CR2, a serine/threonine-rich region, links CR1 and CR3 and contains a 14-3-3 scaffold binding site. CR3 (residues 457–717) (Zaman et al., 2019), near the C-terminus, is a kinase-binding domain that activates upon phosphorylation, comprising a glycine-rich ATP-phosphate binding lobe (residues 462–469) and a longer C-terminal lobe (residues 593–623) (Śmiech et al., 2020; Maloney et al., 2021). The most common mutation, V600E, is situated in the CR3 region of *BRAF*, disrupting its autoinhibition mechanism, hence contributing to oncogenic activity due to destabilization within the inactive form of *BRAF* (Park et al., 2019).

In our secondary structure analysis, we predicted that *BRAF* V600E falls into a loop structure identified by Park et al. (2019), as the inhibitory loop (Park et al., 2019). Loops are one important determinant in the stability and functions of proteins. Superimposing *BRAF* V600E on its wild-type counterpart gave a high RMSD value of 1.7216; DUET thermodynamic stability analysis also predicted that this mutation would highly be destabilizing, with a value of −2.118 kcal/mol. The results predict that this missense variant will have a profound impact on the structure of the protein and severely disrupt normal function. Computational binding assays for Encorafenib with *BRAF* wild-type and its V600E variant in Figure 3 showed differential binding behavior. The binding energy of the wild-type-Encorafenib complex was −5.915 kcal/mol, displaying two hydrogen interactions at D742 and A762. By contrast, V600E-Encorafenib had only one hydrogen bond interaction at Y746 shown with a binding energy of −3.509 kcal/mol for the conformational pose. With this low binding energy score with two hydrogen bond interactions in the case of a wild-type-Encorafenib complex, it indicates that the wild-type protein binds with the inhibitor very tightly and, hence, is effective under normal conditions. The V600E-Encorafenib has a higher binding energy score and, therefore, a weaker binding affinity, though it is still predicted to be able to inhibit its target protein. Both complexes interacted in proximity to the residues expected to interact with the inhibitors near the C-tail inhibition binding sites, F743 and A749, respectively (Martinez Fiesco et al., 2022), and both complexes interacted nearby. Our results indicate that Encorafenib may have a reduced efficacy in V600E variant proteins. The lower inhibition ability of Encorafenib in patients with the V600E variant could be a factor explaining why these patients may not respond to monotherapy, and often require a combination with other drugs such as Cetuximab (an EGFR inhibitor), and Binimetinib a (MEK inhibitor) to gain benefit from the treatment. It is because blocking the BRAF activity causes an over activation of EGFR, the latter must also be inhibited to gain a better response (Kopetz et al., 2019). Therefore, Encorafenib might be better at targeting wildtype BRAF proteins whether blocking this member of the pathway is needed in designing a new drug regimen for patients without the V600E variant. *KRAS* and *NRAS* are gene homologs belonging to the RAS superfamily, which play crucial roles in the RAS-RAF-MEK-MAPK signaling pathways (Fernández-Medarde and Santos, 2011) and PI3K-AKT-mTOR pathways (Castellano and Downward, 2011). Approximately 19% of cancer patients develop a RAS mutation, including approximately 3.4 million new cases yearly that are positive for RAS. Moreover, 75% of mutated RAS isoforms are *KRAS* (Prior et al., 2020). *KRAS* mutations occur in 11.6% of all cancer types, as per TCGA data. They are mostly prevalent in pancreatic ductal adenocarcinoma (PDAC) (81.72%), CRC (37.97%), and non-small cell lung cancer (NSCLC) (21.20%) (Yang et al., 2023). In mCRC patients, ∼60% have *KRAS* mutations (Peeters et al., 2015), primarily in codons 12, 13, 61, 117, and 146 (Serebriiskii et al., 2019). Over 90% of *KRAS* mutations occur at codons 12 and 13, part of the p-loop of the GTP binding domain, with codon 12 being more associated with oncogenesis and higher mortality than codon 13 (Colussi et al., 2013). The most prevalent *KRAS* mutations in CRC are G12D (28.04%), followed by G12V (18.50%) and G13D (18.10%), according to http://cBioPortal.org (Yang et al., 2023). Among the 86 samples in our study, 18.60% carried the G12D mutation, 5.81% carried the G12V mutation, and 3.48% carried the G13D, G12A, and G138E mutations. Additionally, 2.32% of samples had K117N and A146T mutations, while 1.16% of samples had G12R, G12S, E49K, or Q61H mutations. Except for E49K and G138E, all identified *KRAS* mutations are within hotspots. *KRAS* mutations have been reported in Saudi Arabia in the past at a frequency of 28.6%–56%, with G12D and G12V being the most prevalent variants (Siraj et al., 2014; Beg et al., 2015; Al-Shamsi et al., 2016; Alfahed, 2023). In comparison, our data demonstrated ∼10% lower incidences of G12D than international and regional studies, which may be related to the limited sample size. Surprisingly, G138E, a rare variant that was only reported in COSMIC twice from large intestine samples, was detected in three cases and thus could be more common in Saudi or Arab CRC patients. One patient carried a rare *KRAS* mutation (E49K), which was concomitantly found with another *KRAS* mutation (G138E). It was reported only twice so far from large intestine samples in COSMIC. CancerVar has classified G12D as Tier I, strong clinical significance, and nine variants as Tier II, potential clinical significance: G12A; G12R; G12S; G12V; G13D; Q61H; K117N; G138E; and A146T. E49K was classified as Tier III with unknown clinical significance. The clinical significance of these variants aligns with expectations, except for G138E and E49K, which lack sufficient clinical data.

In the cell, *KRAS* is expressed in two isoforms (*KRAS*-4B and *KRAS*-4A) due to alternative splicing of the exon 4. The term *KRAS* refers to the *KRAS*-4B transcript, as it is more highly expressed than *KRAS*-4A (Nuevo-Tapioles and Philips, 2022). The *KRAS* gene, located at chromosome 12p11.1-12p12.1, encodes a protein of 188 amino acids, divided into five exons (Huang et al., 2021). *KRAS* is a cascade signaling protein that is activated by GTP and deactivated by GDP, a process that needs to be balanced for normal protein function (Zhu et al., 2021). *KRAS* and *NRAS* share two main conserved domains. The GTP-binding (G) catalytic domain (residues 1–165) is highly conserved and identical in both RAS forms. It comprises two lobes: the effector lobe (residues 1–86) contains switch 1 (S1) (residues 30–38), switch 2 (S2) (residues 60–76), and the phosphate-binding region (P-loop) (residues 10–17), all essential for GTP binding. The allosteric lobe (residues 87–166) is less conserved, with 70%–80% similarity. The hypervariable region (HVR) (residues 167–188) at the C-terminus is the least conserved and crucial for post-translational modifications for membrane anchoring (Jancík et al., 2010; Zeitouni et al., 2016; Pantsar, 2020; Han et al., 2021; Nuevo-Tapioles and Philips, 2022). Our identified mutations in codons 12 and 13 (G12A, G12D, G12R, G12S, G12V, and G13D) are in the P-loop region of the G domain. These mutations disturb the active site’s geometry, preventing GTP hydrolysis and locking the RAS protein in its active state (Malumbres and Barbacid, 2003). The rare E49K and the Q61H mutations, located in exon 3, affect the G domain; Q61H is in the S2 region, potentially disrupting GTPase activity by blocking GTP transition to GDP. Mutations K117N, G138E, and A146T are in exon 5, near the HVR. K117N and A146T, found in hotspot codons, are known to predict resistance to Cetuximab plus Irinotecan (Loupakis et al., 2009). Codon 146 mutations occur in ∼4% of CRCs and are more common than codon 61 mutations (Edkins et al., 2006). Functional studies on Q61H and K117N show increased proliferation and high levels of GTP-bound *KRAS*, indicating they affect GTPase activity by maintaining the active form (Stolze et al., 2015). G138E is a rare mutation located near the HVR with no current functional data. Secondary structure analysis of *KRAS* variants revealed all variants were in loop regions except E49K, which is on a ß-pleated sheet. No obvious changes were seen in secondary structure elements, except an increased loop size in the G138E mutant (Supplementary Figure 1). Loop regions are important for protein stability, and mutations can disturb this stability. E49K showed no obvious effects. Superimposing *KRAS* mutant and wild-type forms revealed significant alterations at affected residues for all variants (Figure 3), with deviations ranging from 1.1634 to 1.5958, except for Q61H and K117N, which showed higher deviations (2.3574 and 2.789, respectively). These deviations likely affect the binding site geometry, particularly at codon 12, where sidechain addition disturbs GTP hydrolysis complex binding. Q61H and K117N significantly disrupt the amino acid structure, altering overall protein function and GTPase activity. Protein stability analysis indicated seven *KRAS* variants to destabilize the structure (G12D, G12R, G12S, G13D, K117N, G138E, A146T). Other four variants further stabilize the structure (G12A, G12V, E49K, and Q61H). The increased or decreased free energy values demonstrate the influence of these mutations on protein stability. A146T and K117N are highly destabilizing, predicting decreased catalytic ability, while Q61H is highly stabilizing. E49K weakly increases stability, demonstrating low impact on function.

*NRAS* is a homolog to *KRAS*, with similar protein structure and function (Colicelli, 2004). *NRAS* mutations occur in 3.03% of all cancers (Consortium et al., 2017), predominantly in skin (10%–25%) (Jakob et al., 2012), acute myeloid leukemia (9.76%) (Consortium et al., 2017), and colon (∼4%) (Cicenas et al., 2017). In CRC, the majority of *NRAS* mutations occur at codons 12, 13, and 61, with codon 61 being the most frequently mutated (Vaughn et al., 2011). In this study, two *NRAS* variants were identified, Q61K and R102Q (2.32%). Previous studies from Saudi Arabia have reported NRAS variants occurring at a rate of 2%–4% (Al-Shamsi et al., 2016; Alharbi et al., 2021; Alfahed, 2023). Our findings align with this but are 2% lower than the international CRC prevalence. The R102Q variant, although present in low VAF, is considered novel and has not been previously reported in CRC. The low VAF suggests it could be an artifact, present in a small subset of cancer cells, or with potential biological relevance (i.e., low- frequency driver mutations arising in later-stage subclones). CancerVar classifies Q61K as Tier II—potential clinical significance, as expected. R102Q is predicted to be benign by CancerVar pathogenicity prediction tools and is classified as a Tier III variant of unknown significance, though our study’s selected tools suggest it is pathogenic. Therefore, its true pathogenicity remains to be functionally tested for confirmation. *NRAS* is located at chromosome 1p13.2 (Mitchell et al., 2008) and encodes a protein of 189 amino acids across seven exons (Eisfeld et al., 2014). The Q61K variant is mapped to the S2 region of exon 3, within the G catalytic domain. Due to this location, it is expected to affect *NRAS* proteins by disrupting GTP hydrolysis, thus activating the protein. R102Q is located in exon 4, also a part of the catalytic domain. Therefore, it is predicted to similarly influence the catalytic site’s conformation. 3D structural modeling showed significant deviations between the variant and wild-type structures of Q61K (4.263) and R102Q (2.4139). Both variants increased protein stability (Q61K: 0.486; R102Q: 0.012). Q61K likely makes the G domain more rigid, altering the binding site, while R102Q’s impact on G domain function cannot be predicted based on our data.

We compared our selected tools with better algorithms against CancerVar tools to make accurate predictions for identified somatic variants. Our pathogenicity prediction tools showed a significant difference against CancerVar tools. Variants such as *KRAS* G12S, E49K, and *NRAS* R102Q were predicted as benign by CancerVar but pathogenic by our tools. The other variants were consistently identified as pathogenic (Tables 2, 3). *KRAS* G12S, located at codon 12, is expected to confer drug resistance due to increased activation. CancerVar’s tools mostly predicted it as benign, while our tools as pathogenic. ROC analysis demonstrated the superior performance of 6/7 (85%) of our selected tools (PrimateAI, BayesDel_addAF, REVEL, FATHMM-MKL, CADD_Phred). Significant z-scores further supported these findings, highlighting tool performance variability. Overall, study-selected tools outperformed CancerVar tools, probably due to the advanced machine learning algorithms these tools use to integrate diverse data sources. The best-performing tools in this study rely on the more complex algorithms such as deep learning, multiple kernel learning, support vector machines, deep neural networks, and advanced Bayesian statistical frameworks (PrimateAI, REVEL, FATHMM-MKL, CADD_Phred, DANN, and BayesDel_addAF, respectively). They also incorporate additional features, such as evolutionary conservation, functional annotations, and population allele frequencies, providing different angles of prediction. By comparison, the CancerVar tools SIFT, FATHMM, and PolyPhen2_HDIV are older and employ simpler algorithms and most likely out-of-date training data. Being foundational models, their much lower complexity may be affecting their accuracy relative to newer tools. The newer tools also have the largest and most diverse training datasets, covering more variant types and contexts. This study emphasizes that advanced and diverse prediction tools, when possible, must be used in combination for comprehensive variant assessment. It is, however, paramount to complement such predictions with clinical or functional evidence for the correct determination of pathogenicity for a somatic variant.

## 5 Conclusion

In conclusion, our study, one of the few from Saudi Arabia, supports pathogenicity for some known variants and predicts the pathogenicity of other rare variants. Functional studies, however, are needed for validation. We reported on the prevalence of somatic variants from CRC patients in a Saudi hospital, identifying 14 variants in actionable genes, including one novel variant (*NRAS* R102Q) and two rare variants (*KRAS* E49K and G138E). *KRAS* G138E, present in three patients, was previously reported only twice from large intestine samples and might be more common in Saudi or Middle Eastern populations. The *KRAS* E49K co-occurred with *KRAS* G138E, while the *NRAS* R102Q co-occurred with *KRAS* G12D. Wild-type *BRAF* proteins showed higher binding affinity to Encorafenib, suggesting more effective inhibition compared to the variant protein V600E. Our genotype-protein-phenotype analyses emphasized the pathogenicity of identified variants, highlighting the necessity of effective targeted therapies. Whether the identified rare or novel variants were functionally shown to be activating their respective proteins, clinical decisions can be made to tailor treatment for patients harboring these variants. The prevalence of somatic variants in Saudi Arabia within the *BRAF*, *KRAS*, and *NRAS* genes is roughly 47.7%, which is consistent with global estimates. As cell cycle modulators, these genes continue to be attractive therapeutic targets. Overall, this study highlights the significance of comprehensive molecular screening and bioinformatics in understanding the mutational landscape of CRC in the Saudi population, with the goal of improving targeted drug therapy.

## Data Availability

The raw data supporting the conclusions of this article will be made available by the authors, without undue reservation.

## Supplementary material

SUPPLEMENTARY FIGURE 1 Secondary structure analysis for BRAF, KRAS, and NRAS variant proteins; **(A–C)**, respectively. For each variant protein, the upper segment of the comparison illustrates the wild-type secondary structure (WT), while the lower segment displays the variant secondary structure.
