# Supplementary figures and images for: Exploring somatic mutations in BRAF, KRAS, and NRAS as therapeutic targets in Saudi colorectal cancer patients through massive parallel sequencing and variant classification
Source: Front Pharmacol. 2024 Nov 20;15:1498295. doi: 10.3389/fphar.2024.1498295 (PMC11614610; doi:10.3389/fphar.2024.1498295)

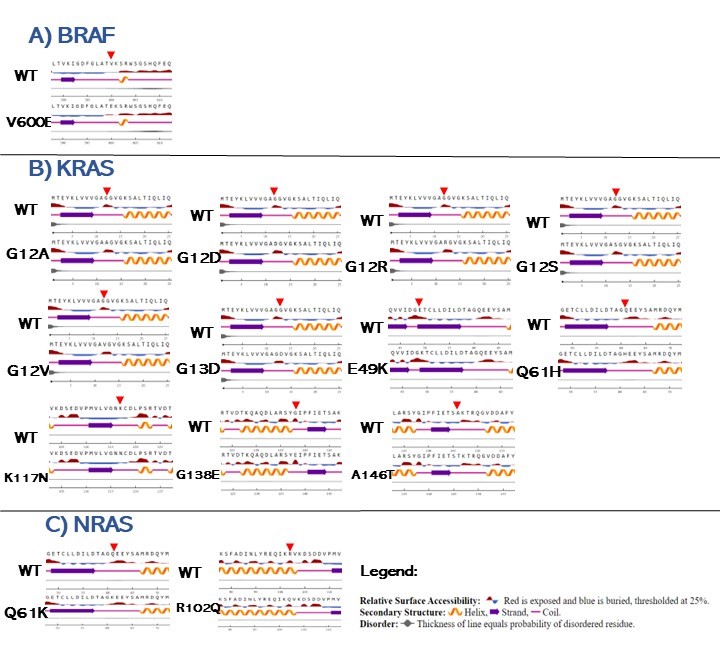

Supplement: Supplementary file 1 [file Image1.JPEG]
